# Alexithymia and somatisation in patients with remitted major depression and their impact on social functioning

**DOI:** 10.4102/sajpsychiatry.v22i1.886

**Published:** 2016-10-21

**Authors:** Hakan Delibas, Asusinem A. Kirdok, Almila Erol

**Affiliations:** 1 Bozyaka Education and Research Hospital, Turkey; 2 Ozel Tinaztepe Hospital, Turkey; 3 Izmir Ataturk Training and Research Hospital, Turkey

## Abstract

**Objectives:**

The aim of the present study is to investigate the level of social functioning, alexithymia and somatisation in patients with major depressive disorder who achieved full remission and to examine the impact of alexithymia and somatisation on social functioning in patients with major depression who are in full remission.

**Methods:**

A total of 117 outpatients with major depression and full remission and 42 healthy controls were included in the study. The participants were administrated Affect Underpinned by Severity and Social Impairment Questionnaire (AUSSI) to evaluate social functioning and depressive symptoms, Toronto Alexithymia Scale (TAS) to evaluate alexithymia and Somatosensory Amplification Scale (SSAS) to evaluate somatisation. Forty-one patients who scored higher or equal to the cut-off score of 5 on the social impairment subscale of AUSSI were classified as having impaired social functioning, whereas 76 patients who scored less than 5 were classified as having unimpaired social functioning.

**Results:**

There were no significant differences between the groups for AUSSI mood symptoms subscale score. Patients with impaired social functioning scored higher than controls on TAS score. Patients with both impaired and unimpaired social functioning scored higher than controls on SSAS scores. The only significant predictor of social impairment in patients with major depression who were in full remission was AUSSI mood symptoms subscale score.

**Conclusion:**

Patients with major depression may still have social impairment after remission. Depressive symptoms are the most important predictors of social functioning in patients with remitted depression. Maximum precautions should be taken to treat depression without leaving any residual symptoms.

## Introduction

Impairments in social functioning are common in major depressive disorder.^1^ Although there is an association between decreases in clinical symptoms and improvements in social functioning during acute treatment of depression, social functioning often does not return to baseline, and long-term impairments in social functioning may persist even in patients whose depression is in clinical remission.^2^ Therefore, social functioning measures may provide a window into patients’ recovery that incompletely overlaps with measures of depressive symptoms, and factors other than depressive symptoms may be responsible for some of the social functioning problems which patients with major depression experience.

Alexithymia is marked by difficulties in verbally describing affect and in differentiating mental states from bodily sensations. It was in the context of psychosomatic research that the alexithymia concept was first introduced, but its use extends beyond the field of psychosomatics now. In fact, alexithymia is associated with higher somatic symptom reporting, and it may be associated with somatisation by way of focusing on or amplifying the somatic sensations associated with emotional arousal or by misinterpreting these as symptoms of disease.^3^ Besides, somatisation has been shown to be associated with both alexithymia and depressive disorders.^4^

Previous research conducted with general population samples suggested that alexithymia may be highly associated with depression.^5^ Consequently, studies on depression showed that alexithymia seems to be related with the severity of depression in patients with major depression, but it also shows relative stability over time.^6^ In addition, there is evidence that alexithymia relates to specific interpersonal problems.^7^ Despite this, neither the associations between social functioning and alexithymia nor the association of alexithymia with impaired social functioning in patients with depression have been studied before.

The aim of the present study is to investigate the level of social functioning, alexithymia and somatisation in patients with major depressive disorder who achieved full remission and to examine the impact of alexithymia and somatisation on social functioning in patients with major depression who are in full remission. We hypothesised that some of the patients with major depression who achieve full remission would still have impairments in social functioning, and those patients who have impaired social functioning would have higher levels of alexithymia and somatisation compared with the patients with unimpaired social functioning and healthy controls.

## Methods

### Subjects and design

A total of 117 patients with ‘major depression, in full remission’ who were being followed up in Bozyaka Education and Research Hospital (Izmir, Turkey) outpatient clinic of psychiatry after achieving full remission were included in the study. The patients who had applied for a follow-up visit between January 01 and June 30, 2014, were subsequently enrolled in the study. All the patients were on antidepressant drugs but free of benzodiazepines and other psychotropic medications. Forty-two healthy controls were also included in the study. Healthy controls were recruited from the hospital cleaning staff and their family members.

Patients were diagnosed using Structured Clinical Interview for Diagnostic and Statistical Manual of Mental Disorders-IV (SCID-I). Patients who met the criteria for ‘major depressive disorder, in full remission’ were included in the study. Patients who did not meet criteria for full remission or who had current co-morbid psychiatric diagnoses were excluded. Healthy controls were screened with SCID-I, and those who met criteria for any current or past psychiatric disorder were excluded. Exclusion criteria for all participants included mental retardation, identifiable neurological disorder or significant medical illness, substance use in the past 6 months and age less than 18 years and more than 65 years. All subjects were required to be literate. Information about these criteria was derived from direct interviews with participants. After description of the study, written informed consent was obtained from all participants.

A total of 166 patients were evaluated for the study. Twenty-nine patients were not in full remission, 12 did not give informed consent, 4 had alcohol abuse, 2 had other substance abuse, 1 had panic disorder and 1 was illiterate and they were not included in the study. Forty-eight controls were evaluated for the study and three who had depressive disorder in the past, one who had bipolar disorder, one who had obsessive-compulsive disorder and one who had alcohol abuse were excluded.

All the participants were given Affect Underpinned by Severity and Social Impairment Questionnaire (AUSSI) to evaluate social functioning and depressive symptoms, Toronto Alexithymia Scale (TAS) to evaluate alexithymia and the Somatosensory Amplification Scale (SSAS) to evaluate somatisation. Forty-one patients who scored above or equal to the cut-off score of 5 on the social impairment subscale of AUSSI were classified as having impaired social functioning, whereas 76 patients who scored less than 5 were classified as having unimpaired social functioning.

The study was approved by the Bozyaka Education and Research Hospital ethics committee.

### Study instruments

**Affect Underpinned by Severity and Social Impairment Questionnaire (AUSSI):** This is an 11-item self-report scale of depression severity, assessing both mood state and social impairment domains and designed to be independent of sub-typing features. It consists of six items that rate the severity of mood symptoms and five items that rate social impairment. Each item is scored on a 0 to 3-pointLikert scale. It allows clinicians to describe mood symptoms and social impairment and to follow-up residual symptoms after treatment in patients with major depressive disorder.^8^

**Toronto Alexithymia Scale-20:** This instrument is a 20-item self-report scale. It consists of three dimensions that indicate the degree of alexithymia: difficulties in identifying feelings, difficulties in describing feelings and externally oriented thinking. Each item is scored on a 5-point Likert scale, with five items negatively keyed. Total scores range from 20 to 100, with higher scores indicating more pronounced alexithymia.^9^

**Somatosensory Amplification Scale (SSAS):** This scale measures how the physical symptoms are experienced by patients and their susceptibility to somatisation. It is applicable to the patients with psychiatric or medical disorders and also to the healthy community. All items are rated on a 1- to 5-point Likert scale and ratings are summed to produce a total score that ranges from 10 to 50.^10^

### Statistical analysis

The gender distribution, marital status and employment status of patient and control groups were compared using chi-square test. Patients with impaired social functioning and patients with unimpaired social functioning were compared via independent-samples test for the number of previous depressive episodes and age at illness onset. One-way analysis of variance (ANOVA) was used to compare age, education level and AUSSI, TAS and SSAS scores of patient groups and controls. Tukey honestly significant difference test was applied in the posthoc analysis for the multiple comparisons of the three groups. Linear regression analysis was used to assess the significant predictors of social impairment measured by AUSSI social impairment subscale.

## Results

Of the patient group, 41 (35.0%) had impaired social functioning, whereas 76 (65.0%) had unimpaired social functioning. Patients with impaired functioning, patients with unimpaired social functioning and controls were comparable for gender distribution, marital status, employment status, age and education level. When it comes to employment status, homemakers, students and the people retired on time were considered in the employed group. The socio-demographic characteristics of patient and control groups are depicted in Table 1.

**TABLE 1:** Socio-demographic characteristics of patients and controls.

Variable	Patients with impaired social functioning (*n* = 41)	Patients with unimpaired social functioning (*n* = 76)	Controls (*n* = 42)	Statistical test
Gender	22F/19M	48F/28M	25F/17M	χ^2^ = 4.23, *p* = 0.10
Marital status	29MA/4S/8D	49MA/11S/16D	29MA/5S/8D	χ^2^ = 0.74, *p* = 0.95
Employment status	32E/9UE	64E/12UE	40E/2UE	χ^2^ = 5.16, *p* = 0.08
Age (mean ± SD)	42.8 ± 9.5	41.1 ± 11.2	40.8 ± 8.6	*F* =1.1, *p* = 0.39
Education (years) (mean ± SD)	7.2 ± 3.9	8.4 ± 4.1	8.5 ± 4.5	*F* = 1.4, *p* = 0.26

F, female; M, male; MA, married; S, single; D, divorced or separated; E, employed; UE, unemployed.

Patients with impaired social functioning and patients with unimpaired social functioning did not differ with regard to the number of previous depressive episodes and age at illness onset. The mean number of previous depressive episodes were 2.7 ± 2.1 in patients with impaired social functioning and 2.6 ± 1.8 in patients with unimpaired functioning (*t* = 0.2, *p* = 0.87). The mean age at illness onset was 37.6 ± 11.0 years in patients with impaired social functioning and 33.8 ± 11.8 in patients with unimpaired functioning (*t* = 1.7, *p* = 0.09).

Differences between groups on scale scores were examined by one-way ANOVAs. There were statistically significant differences between groups on AUSSI social impairment subscale scores (*F* = 104.1, df = 2, 156, *p* < 0.001), while there were no significant differences between the groups for AUSSI mood symptoms subscale score (*F* = 2.9, df = 2, 156, *p* = 0.06). There were statistically significant differences between the groups for both TAS and SSAS scores (*F* = 4.1, df = 2, 156, *p* = 0.02, and *F* = 6.9, df = 2, 156, *p* = 0.001 respectively). Tukey HSD was applied in the posthoc analysis. As expected, patients with impaired social functioning scored higher than both the patients with unimpaired social functioning (*p* < 0.001) and controls (*p* < 0.001); patients with unimpaired social functioning scored worse than the controls (*p* = 0.001) on the AUSSI social impairment subscale. Patients with impaired social functioning scored worse than controls on the TAS score (*p* = 0.02), but there were no significant differences between patients with unimpaired social functioning and controls, and between patients with impaired and unimpaired functioning. On SSAS scores, patients with impaired and unimpaired social functioning both scored higher than controls (*p* = 0.001 and *p* = 0.03, respectively), whereas there were no statistically significant differences between the two patient groups. Comparison of patients with impaired social functioning, patients with unimpaired social functioning and controls for AUSSI, TAS, and SSAS scores are depicted in Table 2.

**TABLE 2:** Comparison of patients and controls for AUSSI, TAS and SSAS scores.

Variable	Patients with impaired social functioning mean ± SD	Patients with unimpaired social functioning mean ± SD	Controls mean ± SD	One-way analysis of variance	Tukey HSD
AUSSI social impairment subscale	7.3 ± 2.4	2.9 ± 2.5	1.5 ± 1.4	*F* = 104.1, *p* < 0.001***	P1 vs. control *p* < 0.001*** P2 vs. control *p* = 0.001** P1 vs. P2 *p* < 0.001***
AUSSI mood symptoms subscale	6.3 ± 3.6	4.6 ± 3.9	4.5 ± 3.7	*F* = 2.9, *p* = 0.06	P1 vs. control *p* = 0.10 P2 vs. control *p* = 0.99 P1 vs. P2 *p* = 0.07
TAS	56.5 ± 14.2	51.7 ± 12.0	48.8 ± 13.0	*F* = 4.1, *p* = 0.02*	P1 vs. control *p* = 0.02* P2 vs. control *p* = 0.49 P1 vs. P2 *p* = 0.11
SSAS	32.4 ± 10.6	29.6 ± 9.1	25.0 ± 8.8	*F* = 6.9, *p* = 0.001**	P1 vs. control *p* = 0.001** P2 vs. control *p* = 0.03* P1 vs. P2 *p* = 0.25

P1, patients with impaired social functioning; P2,patients with unimpaired social functioning; AUSSI, Affect Underpinned by Severity and Social Impairment Questionnaire; TAS; Toronto Alexithymia Scale-20; SSAS; Somatosensory Amplification Scale.

* *p* < 0.05;

** *p* < 0.01,

*** *p* < 0.001.

Multiple stepwise linear regression analysis was used in order to determine the variables that predict social functioning in patients with major depression, full remission. Only patient groups were included in this analysis. AUSSI social impairment subscale score was entered as the dependent variable; age, education level, number of previous depressive episodes, age at illness onset, AUSSI mood symptoms subscale score, TAS and SSAS scores were entered as the independent variables. Only AUSSI mood symptoms subscale score was the significant predictor of social impairment. It explained 12.8% of the variance in AUSSI social impairment subscale score (*p* < 0.001).

## Discussion

In our study, we examined patients who had major depression and found out that 35% of the patients had impaired social functioning, although they were in full remission. This was in line with previous research, which has shown that 30% to 60% of patients with depression had impairments in social functioning^11^ and confirmed our first hypothesis that some of the patients with major depression would still have social impairments, even after they achieved full remission.

Although the number of previous depressive episodes and age at illness onset were slightly higher in patients with impaired social functioning in comparison to patients with unimpaired social functioning, these differences were not statistically significant. Nevertheless, to our knowledge, no studies have shown an effect of age at illness onset or number of depressive episodes on social adjustment. Despite this, patients with chronic depression seem to have worse social outcomes than patients with episodic depression independent of the number of depressive episodes.^12^ Therefore, our findings are not likely to be a result of low statistical power.

We used AUSSI social impairment subscale cut-off point to form two groups of patients with impaired and unimpaired social functioning. Thus, as expected, patients with impaired social functioning had higher scores of social impairment. Moreover, they also had higher social impairment scores than the controls. Interestingly, patients also defined to have unimpaired social functioning had higher social impairment scores than healthy controls. This indicates that overall sample of depressive patients had poorer social functioning compared to healthy controls who had never had a depressive episode in the past. Given the association of depression with impairment of social functioning even after remission, this is an expected finding that is in line with previous research.^2^

With regard to mood symptoms, there were no statistically significant differences between the three groups. Again, this is an expected finding, as we included patients who were in full remission. Yet, the patients with impaired social functioning had higher levels of alexithymia and somatisation compared to healthy controls. This provides evidence that alexithymia and somatisation may have influence on social functioning independent of their interaction with mood symptoms. Nevertheless, findings of previous research show that alexithymia relates to various interpersonal problems, and interpersonal relationships have important associations with social functioning.^7^ Therefore, we performed a regression analysis involving both patient groups to find out significant predictors of social functioning in depression and also to find out whether alexithymia and somatisation had an impact on social functioning, independent of their interaction with mood symptoms and other illness confounders, such as age at illness onset and number of depressive episodes. Contrary to our expectations, we found out that neither alexithymia nor somatisation were among significant predictors of social functioning, and mood symptoms were the only significant predictor of social functioning in remitted depressive patients.

In this study, we made the diagnosis of ‘major depressive disorder, in full remission’ using SCID-I. Full remission in major depression is conceptualised as a period during which an improvement of sufficient magnitude is observed that the individual is asymptomatic (i.e. no longer meets syndromal criteria for the disorder and has no more than minimal symptoms).^13^ An operational criterion of scoring 7 or less on the 17-item Hamilton Depression Rating Scale was also proposed.^13^ Our sample of patients who were in full remission according to SCID-I also had some minimal symptoms of depression measured by AUSSI mood symptoms subscale. Previous research shows that depressive symptoms have strong relationships with social adjustment, and depressive symptom severity predicts poorer social functioning.^14^ In spite of the fact that all the patients in our sample were in full remission, and just had minimal symptoms, mood symptoms were still the only significant predictor of social impairment. Our findings support and expand the findings of previous studies bringing out evidence that even minimal symptoms of depression can impact social functioning. This also brings up the question whether definition of remission in depression needs to be more rigorous.

On the other hand, alexithymia and somatisation do not appear to predict social functioning independent of their interaction with mood symptoms. As a matter of fact, there is evidence that alexithymia is related to depressive symptom severity and negative mood.^15^ Although mood symptoms explained only 12.8% of the variance in social impairment in this study, different confounders other than depressive symptoms could be responsible for the unexplained variance in social functioning. Certainly, depressive symptoms explain a great deal of the variance, and they are the most important predictors of social functioning in depression in any case.

Our study has some limitations. Firstly of all, it was cross-sectional and the longitudinal changes in social functioning could not be observed. Secondly, our sample size was small. New studies with larger sample sizes are needed to clarify whether alexithymia and somatisation interfere with social functioning in patients with depression. New studies should also search for other potential confounders that may interact with social functioning and may potentially explain the gap between improvement in depressive symptoms and improvement in social functioning. On the other hand, to our knowledge, this is the first study to investigate the associations of alexithymia and somatisation with social functioning in patients with depression.

## Conclusion

Depressive symptoms are the most important predictors of social functioning in patients with depression, even after remission. Therefore, maximum precautions should be taken to treat depressive symptoms totally and without leaving any residual symptoms. It is obvious that syndromal remission do not always end up with returning to baseline functioning levels. In patients with major depression, following up the improvements in daily functioning, along with symptom severity, will definitely be more useful to achieve improvements in social adjustment.
